# An Unusual Case of Penile Spindle Cell Malignancy

**DOI:** 10.7759/cureus.3121

**Published:** 2018-08-08

**Authors:** Das Sri Aurobindo Prasad, Chellappa Vijayakumar, Sathasivam Sureshkumar, Debdutta Basu

**Affiliations:** 1 Surgery, Jawaharlal Institute of Postgraduate Medical Education and Research (JIPMER), Puducherry, IND; 2 Surgery, Jawaharlal Institute of Postgraduate Medical Education and Research, Puducherry, IND; 3 Pathology, Jawaharlal Institute of Postgraduate Medical Education and Research (JIPMER), Puducherry, IND

**Keywords:** penis, immunopathology, sarcomatoid carcinoma

## Abstract

Sarcomatoid carcinomas are unusual high-grade tumors, predominantly composed of spindle cells. There appears to be no predilection for any specific site. A few cases of sarcomatoid tumors arising in the penis have been reported. We report a 60-year-old man with a pathological diagnosis of sarcomatoid carcinoma of the penis. This case reiterates the importance of including sarcomatoid carcinoma in the differential diagnosis, especially at uncommon sites.

## Introduction

The incidence of penile cancer in the Indian subcontinent is 1.8 per 100,000 populations [1]. Almost 95% of these tumors are squamous cell carcinomas (SCC) [1]. Sarcomatoid SCC of the penis is a rare variant of penile cancer, representing only 1–2% of penile carcinomas [2]. These high-grade tumors predominantly composed of spindle cells [3,4]. Sarcomatoid SCC of the penis is a subtype of SCC with a poor prognosis because of its wide hematogeneous spread. It is often difficult to diagnose since it requires additional immunohistochemical (IHC) stains. Only a few cases of sarcomatoid SCC of the penis have been reported so far.

## Case presentation

**Figure 1:**
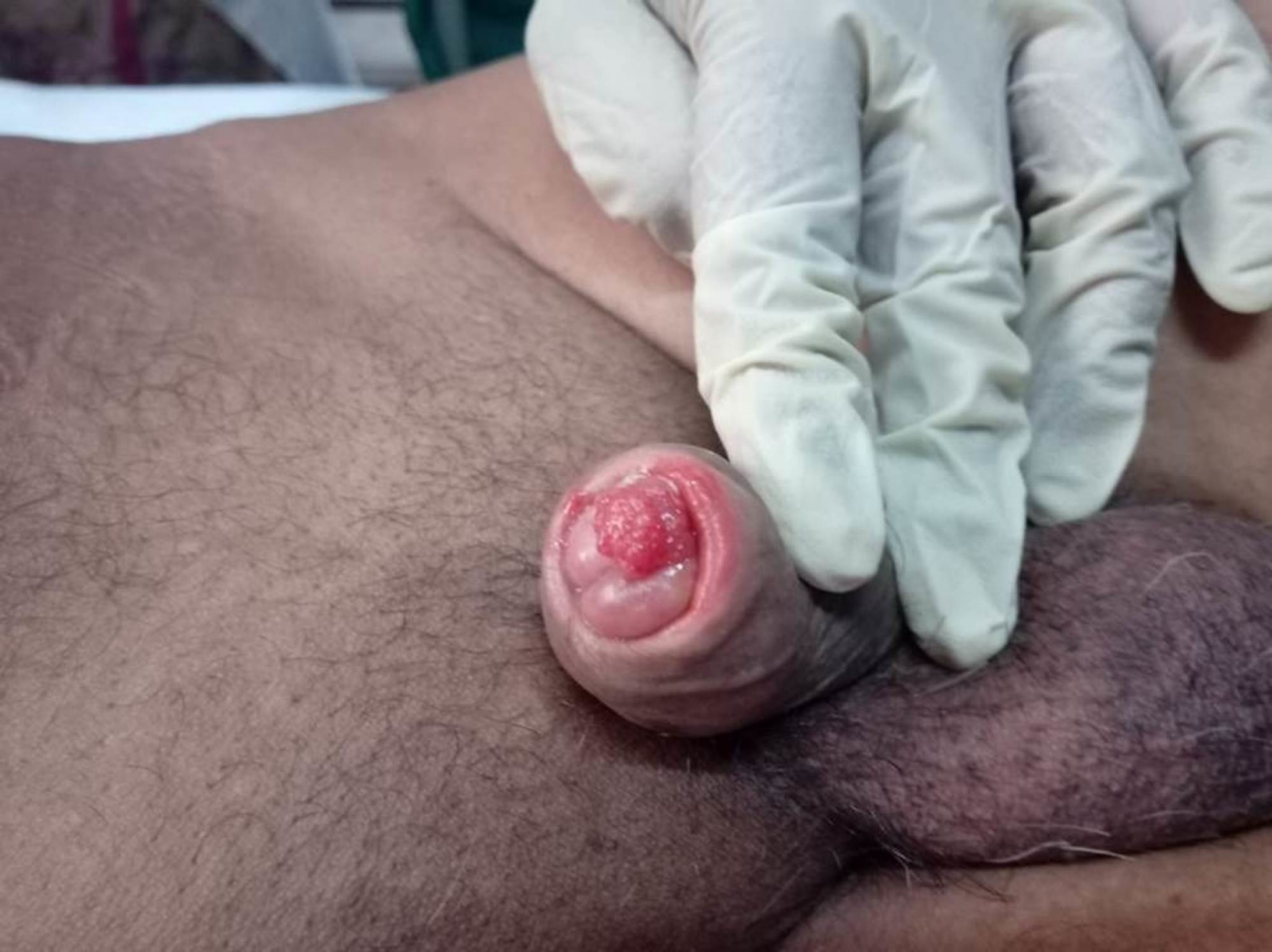
Ulcerated tumor involving the ventral aspect of the glans penis.

A 60-year-old male presented with growth over the glans penis for two months associated with pain. There was no history of contact bleeding. The patient was not circumcised. He had noticed a swelling on the scrotum for the past six months. On examination, there was a 2 x 3 cm ulceroproliferative growth over the glans extending over the corona onto the shaft of penis. The ulcer was fixed to the ventral aspect of glans penis with induration proximally extending up to mid shaft of penis (Figure 1).

Bilateral inguinal lymph nodes were palpable, 3-4 on each side, 0.5 cm in size, firm and mobile in nature. The patient also had a right-sided primary vaginal hydrocele. A clinical diagnosis of SCC of penis was made and a wedge biopsy done. Histopathological analysis revealed an ulcerated stratified squamous epithelial lining with underlying lesion composed of fascicles of spindle cells with moderate pleomorphism. These were very few in number to proceed with IHC stains. A possibility of a primary sarcoma of penis was suggested.

Fine needle aspiration cytology (FNAC) was done from bilateral inguinal nodes which revealed reactive hyperplasia. All blood parameters, liver and renal function tests were normal. The chest X-ray and ultrasound abdomen were unremarkable.

Total penectomy with perineal urethrostomy was performed. The hydrocele was aspirated under aseptic precautions, three days before surgery. Post-operative course was uneventful. The patient was discharged on the eighth post-operative day and was advised to follow up with the regional cancer center for further management.

**Figure 2:**
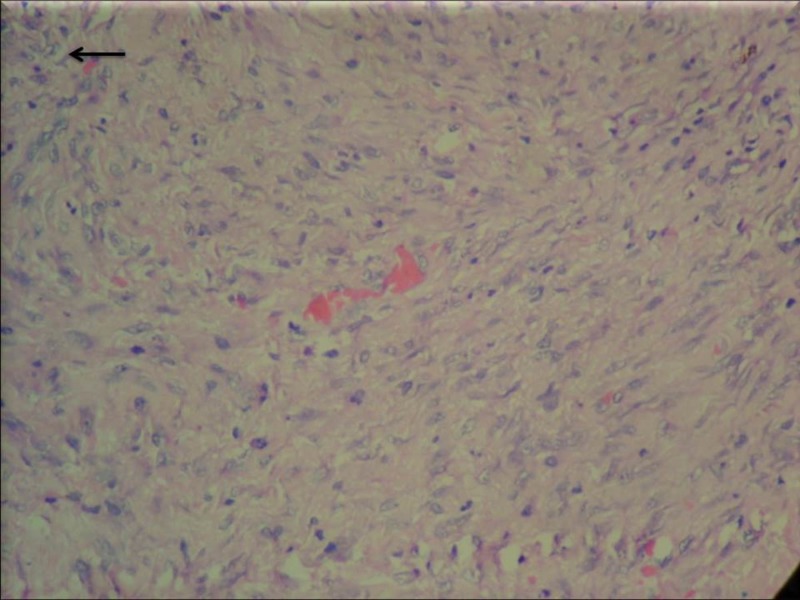
Nests and sheets of spindle cells with moderate nuclear atypia (black arrow) - H&E X200. H&E: Hematoxylin and Eosin

The final histopathological examination revealed moderate pleomorphic spindle cells arranged in sheets of fascicles, the overlying squamous epithelium was dysplastic. Focal areas of necrosis were seen. No definite feature of keratinization was seen (Figure 2).

**Figure 3:**
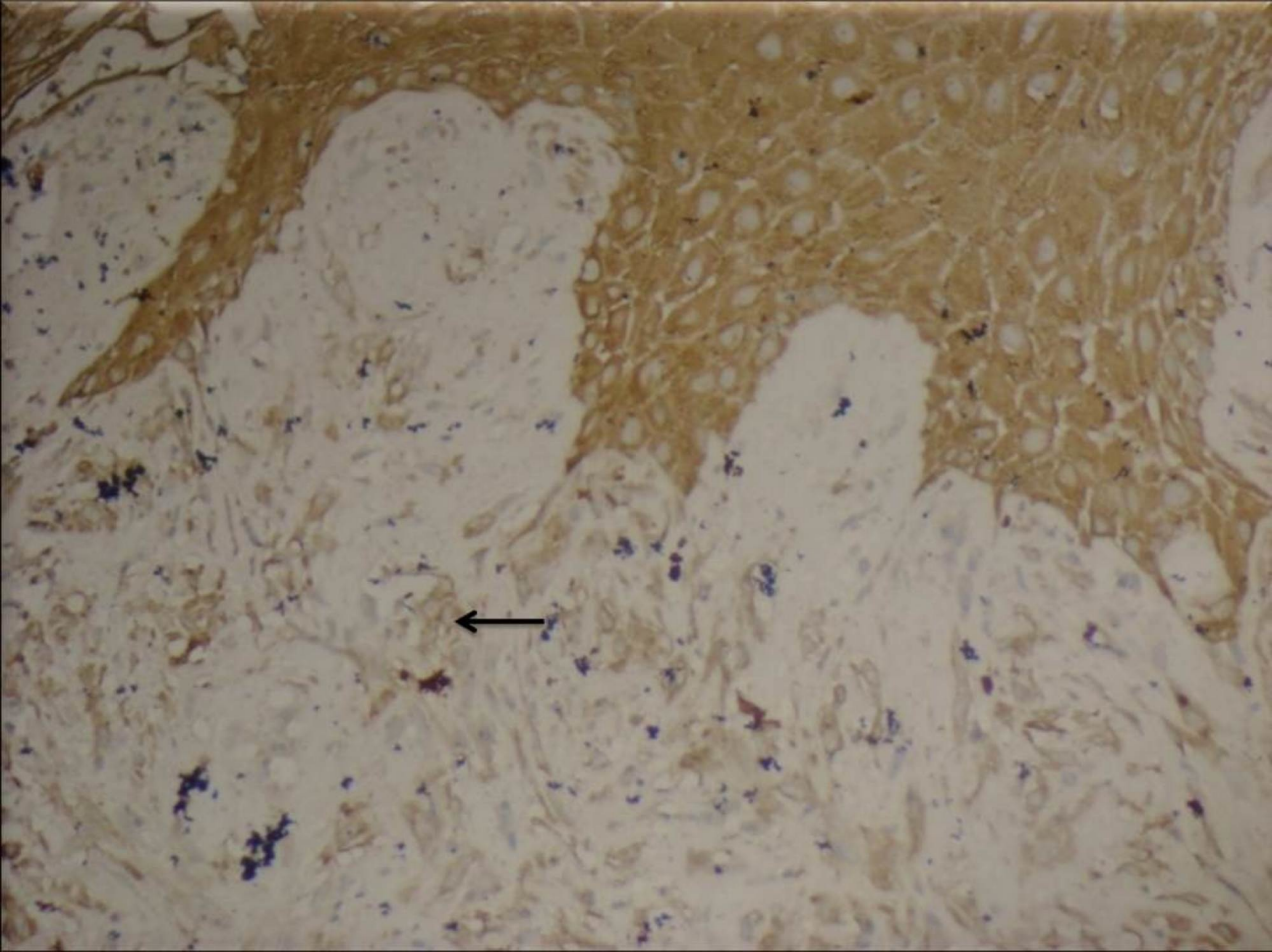
Sarcomatoid areas showing immunoreactivity to pancytokeratin, extending from the overlying stratified squamous epithelium (black arrow) - IHC X 200. IHC: Immunohistochemistry

The tumor cells were strongly positive for pancytokeratin and vimentin, and negative for epithelial membrane antigen (EMA), neuron-specific enolase, CD 34, S100, Melan A and HMB 45 (Figure 3).

The final report was sarcomatoid carcinoma (spindle cell carcinoma) of penis involving the glans. The urethra, proximal resected skin and shaft were free of tumor.

## Discussion

Sarcomatoid SCC is unusual, large and aggressive tumors associated with high rate of lymph node metastasis and poor outcome [2]. The patient presented with an ulceroproliferative lesion over the glans which was initially diagnosed as SCC of the penis. He also had bilateral inguinal nodes, fine needle aspiration of which showed reactive hyperplasia. The initial wedge biopsy was reported as primary sarcoma of penis.

The microscopic diagnosis of sarcomatoid SCC of the penis can be difficult. Since dysplastic changes or carcinoma in situ (CIS) in the overlying epithelium is often absent and/or microscopic examination of the epithelium is hampered due to ulceration, small biopsies are usually inadequate to establish the correct diagnosis. Moreover, small biopsies may consist solely of atypical spindle cells. All this may lead to an erroneous diagnosis of sarcoma, as in this case. The final biopsy was reported as sarcomatoid SCC.

In its classical form, sarcomatoid SCC consists of biphasic pattern with areas of pleomorphic spindle cells admixed with a readily recognizable SCC component, often located in the superficial slices of the tissue. The differential diagnosis of sarcomatoid SCC includes Kaposi sarcoma, Leiomyosarcoma, Angiosarcoma and (spindle cell) Amelanotic melanoma [3].

Controversy still exists about the exact histogenesis of the spindle cell component in sarcomatoid carcinoma, irrespective of its anatomical site. This explains why this type of carcinoma has been referred to by varied terminologies, such as Carcinosarcoma, Spindle cell carcinoma, Pseudosarcoma and Metaplastic carcinoma [4]. However, most authors believe that the sarcomatoid component develops from the carcinomatous areas by metaplasia or dedifferentiation, or more precisely by a premature block in differentiation towards a squamous phenotype. Based on this hypothesis, the two components are considered to originate from the same stem cell [5, 6].

## Conclusions

Sarcomatoid SCC is a rare malignancy of the penis and can present with clinical features of conventional SCC. It is difficult to differentiate clinically from conventional malignancy. Histopathological examination along with IHC stains offers the definitive diagnosis.
